# Cerebrospinal Fluid Amino Acid Profiling of Pediatric Cases with Tuberculous Meningitis

**DOI:** 10.3389/fnins.2017.00534

**Published:** 2017-09-26

**Authors:** Shayne Mason, Carolus J. Reinecke, Regan Solomons

**Affiliations:** ^1^Faculty of Natural Sciences, Centre for Human Metabolomics, North-West University, Potchefstroom, South Africa; ^2^Department of Pediatrics and Child Health, Faculty of Medicine and Health Sciences, Stellenbosch University, Tygerberg, South Africa

**Keywords:** gas chromatography-mass spectrometry (GC-MS), tuberculous meningitis (TBM), pediatric, cerebrospinal fluid (CSF), amino acid profiling

## Abstract

**Background:** In Africa, tuberculosis is generally regarded as persisting as one of the most devastating infectious diseases. The pediatric population is particularly vulnerable, with infection of the brain in the form of tuberculous meningitis (TBM) being the most severe manifestation. TBM is often difficult to diagnose in its early stages because of its non-specific clinical presentation. Of particular concern is that late diagnosis, and subsequent delayed treatment, leads to high risk of long-term neurological sequelae, and even death. Using advanced technology and scientific expertise, we are intent on further describing the biochemistry behind this devastating neuroinflammatory disease, with the goal of improving upon its early diagnosis.

**Method:** We used the highly sensitive analytical platform of gas chromatography-mass spectrometry (GC-MS) to analyze amino acid profiles of cerebrospinal fluid (CSF) collected from a cohort of 33 South African pediatric TBM cases, compared to 34 controls.

**Results:** Through the use of a stringent quality assurance procedure and various statistical techniques, we were able to confidently identify five amino acids as being significantly elevated in TBM cases, namely, alanine, asparagine, glycine, lysine, and proline. We found also in an earlier untargeted metabolomics investigation that alanine can be attributed to increased CSF lactate levels, and lysine as a marker of lipid peroxidation. Alanine, like glycine, is an inhibitory neurotransmitter in the brain. Asparagine, as with proline, is linked to the glutamate-glutamine cycle. Asparagine is associated with the removal of increased nitrites in the brain, whereas elevated proline coincides with the classic biochemical marker of increased CSF protein in TBM. All five discriminatory amino acids are linked to ammonia due to increased nitrites in TBM.

**Conclusion:** A large amount of untapped biochemical information is present in CSF of TBM cases, of which amino acid profiling through GC-MS has potential in aiding in earlier diagnosis, and hence crucial earlier treatment.

## Introduction

Tuberculosis (TB), caused by *Mycobacterium tuberculosis* (Mtb), is an ancient, persistent disease that remains a huge, deadly issue to this day. According to the World Health Organization (WHO, 2016), during 2015 there were an estimated 10.4 million new (incident) TB cases worldwide, of which 5.9 million were men, 3.5 million were women, and 1 million were of children. Six countries accounted for 60% of the new cases: India, Indonesia, China, Nigeria, Pakistan, and South Africa. An estimated 1.8 million people died from TB in 2015, with the mortality of cohorts of more than 100 individuals with extreme drug-resistant (XDR) TB being highest (>40%) in India and South Africa. The incidence of TB in South Africa (population 55 million) for 2015 was estimated to be 454,000 (294,000–649,000) individuals, of whom 33,000 (21,000–44,000) were children (<14 years of age). Hence, South Africa is one of the worst-stricken regions under the scourge of the *M. tuberculosis* bacillus, which depends on humans for its transmission.

TB is most commonly known in its pulmonary form; however, Mtb is not only localized in the lungs but, because of the systematic spread of the tubercule bacilli, can lead to extrapulmonary forms. A preferred site for Mtb—with high blood flow and oxygen content—is the brain; the pathogen is capable of crossing the blood-brain barrier and entering the meninges, which consist of three membranes between the skull and the brain. Infection within the meninges by Mtb leads to tuberculous meningitis (TBM)—the most severe manifestation of TB. The pathology that occurs in the central nervous system (CNS) is similar to that of its pulmonary type, in that lesions (turberculomas) form and can rupture—releasing inflammatory markers. CNS-TB represents up to an estimated 10% of all forms of extra-pulmonary TB (and 1% of total TB) cases (Rock et al., 2005; Bhigjee et al., 2007; Cherian and Thomas, 2011), of which TBM is the principal manifestation.

The gold standard for diagnosis of TBM requires collection of cerebrospinal fluid (CSF), typically through a lumbar puncture. In 2010, Marais et al. proposed a uniform research case definition of TBM based on CSF: (1) TBM could be classified as “definite” when CSF demonstrated acid-fast bacilli on microscopy, a positive Mtb culture and/or passed a positive CSF Mtb commercial nucleic acid amplification test in an individual with symptoms or signs suggestive of the disease. (2) TBM could be classified as “probable” according to a scoring system based on clinical, CSF and neuroimaging criteria, as well as evidence of extraneural TB. In practice, however, TBM is difficult to diagnose in its early stages due to its non-specific clinical presentation. A particular concern is that late diagnosis, and subsequent delayed treatment, leads to high risk of long-term neurological sequelae, and even death.

The two primary biochemical markers currently considered for differential diagnosis of TBM are CSF protein and glucose levels (Solomons et al., 2015). Protein levels are defined as being either elevated—greater than 40 mg/dl (lower cut-off), or significantly elevated—greater than 100 mg/dl (higher cut-off) (Youssef et al., 2006; Hristea et al., 2012). Depressed glucose is defined as <2.2 mmol/l as an absolute value, or relatively as <0.5 CSF:blood glucose ratio as standardized cut-off values (Marais et al., 2010; Solomons et al., 2015).

Our group recently conducted an untargeted proton magnetic resonance (^1^H-NMR) metabolomics study on CSF from 17 pediatric cases with TBM (Mason et al., 2015), which revealed lowered glucose (as expected) and highly elevated lactate as the most defining biochemical markers of the disease, along with perturbed amino acids. The presence of highly elevated CSF lactate was further examined (Mason et al., 2016) and shown to be produced only by the host (with zero contribution from invading Mtb)—an interesting outcome in that the human brain mass produces lactate during a neuroinflammatory disease such as TBM. Indeed, CSF lactate has gained much attention recently in neuroenergetics (Mason, 2017), perhaps as a consequence of being an especially important biochemical marker of Mtb infection. Interestingly, the most prominent CSF metabolites identified by our untargeted ^1^H-NMR metabolomics study as indicators of TBM were also derived in a completely independent study using exactly the same NMR data set and a novel nonparametric classification system (van Reenen et al., 2016)—a method of mathematical modelling for deductive verification of the AMLS (astrocyte–microglia lactate shuttle) hypothesis.

A secondary CSF biosignature of TBM revealed by our previous untargeted NMR metabolomics study consisted largely of perturbed amino acids—alanine, branched-chain amino acids (leucine, isoleucine, and valine), and lysine. These gluconeogenic amino acids, together with ketones, were postulated to be involved in directing (through shuttling mechanisms) CSF lactate, produced by glycolysis in astrocytes, from neurons preferentially into activated microglia in an attempt to aid the eradication of invading Mtb bacilli. Collectively, this biosignature led us to postulate the astrocyte–microglia lactate shuttle. Here, we now report on a more detailed targeted metabolomics study on the amino acid profiles in TBM from a larger collection of CSF samples.

Perturbations in amino acids in TBM cases are not a novel discovery as they have been observed previously in biochemical studies. In 1981, Corston et al. examined eleven viral meningitis and four TBM cases and reported that total amino acid concentrations in CSF were markedly higher in TBM than in viral meningitis. In 1998, Qureshi et al. compared several neurochemical markers in CSF between 11 cases of viral meningitis and 12 of TBM. These authors, using high-pressure liquid chromatography, reported significantly increased aspartic acid, glutamic acid, GABA, glycine, and tryptophan in all cases, whereas only TBM cases exhibited significantly elevated phenylalanine, arginine, and homocysteine. From their results, Qureshi et al. postulated that inflammatory changes in meninges may interfere with amino acid transport across the blood–brain barrier, and proposed several therapeutic approaches. These earlier results on perturbed amino acids in TBM were, however, based on small sample sizes.

Here, we present the first report on a detailed amino acid profiling study of CSF obtained from a cohort of 33 TBM pediatric cases, compared to 34 controls. The method of analysis used was the sensitive platform of gas chromatography-mass spectrometry (GC-MS). Based upon stringent statistical analyses and quality control measures, five amino acids—alanine, asparagine, glycine, lysine, and proline—were identified as being significantly increased in our group of TBM patients, of which we discuss the biological implications in the context of TBM. These observations justify the need for a comprehensive study on biochemical markers for TBM to be validated in a large-scale follow-up investigation.

## Methods

### Sampling

This study was focused on a pediatric group with suspected TBM from the region surrounding Tygerberg Hospital in the Western Cape province of South Africa—an area endemic for TB. A specialized pediatric neurology unit within the hospital focuses on diagnosing and treating cases of TBM in the region. For the purposes of this study, a diagnosis of TBM was based on the uniform research case definition of Marais et al. (2010). Only children (>3 months and <13 years of age) with “definite” and “probable” TBM were included in the experimental patient group (*n* = 33). Our controls (*n* = 34) were age-matched pediatric patients suspected of meningitis, but later confirmed to be meningitis negative. Detailed clinical information on the study cohort is included in Supplementary Information (Table S1). Informed and written consent was obtained from each patient's caregiver and assent if the child was older than 7 years and competent to do so. The study was approved by the Human Research Ethics Committee of Stellenbosch University, South Africa (study no. N11/01/006).

### Gas chromatography-mass spectrometry

Each CSF sample was filtered to remove bacteria—to ensure safety while handling—as well as for the removal of proteins and other macromolecules. Filtration was done using the Sartorius Centrisart®1 10-kDa centrifugal unit by centrifugation of CSF samples for 15 min at 3,000 rpm. Samples were prepared using the commercially available EZ:faast™ analysis kit (Phenomenex) for the analysis of free (physiological) amino acids by GC-MS. See SI for full details on sample preparation, as well as instrument details.

In conjunction with the CSF samples, several external quality control (EQ) samples were included—commercial lyophilized human serum samples spiked with known concentrations of amino acids (Fowler et al., 2008). Calibration standard (CS) samples—commercial standard mixtures of amino acids as part of the EZ:faast™ analysis kit, were also included. The CS samples were used to quantify each block of samples (e.g., a calibration curve was created using CS2 and CS3 and this curve was used to quantify samples in block ➀, CS5 and CS6 were used to calibrate EQ3, and so on). Each block of samples consisted of either seven or eight randomized experimental samples, with a total of 10 blocks. The experimental run was as follows:

Blank||CS1|EQ1A|EQ1B|CS2|➀|CS3|EQ2|CS4|➁|CS5|EQ3|CS6|➂|CS7|EQ4|CS8|➃|CS9|EQ5|CS10|➄|CS11|EQ6|CS12|➅|CS13|EQ7|CS14|➆|CS15|EQ8|CS16|➇|CS17|EQ9|CS18|➈|CS19|EQ10|CS20|➉|CS21|EQ11|EQ1C|EQ1D|CS22||end

### Data analysis

The GC-MS data were deconvoluted, identified and annotated using NIST spectral libraries, and quantified using AMDIS (Automated Mass Spectral Deconvolution and Identification System). These data were amalgamated into one (*n* × *m*) data matrix composed of *m* amino acid identities and *n* sample identities. Each entry consisted of a concentration value calculated in micromoles per liter (μmol/l). Statistical analyses were performed using The Unscrambler® X (V10.4, CAMO software AS, Norway) and the online metabolomics suite, Metaboanalyst 3.0 (www.metaboanalyst.ca) (Xia et al., 2015). Unsupervised principal component analysis (PCA) and Hotelling's *T*^2^-test, with a confidence level of 95%, were used to remove case outliers—in two TBM and two control cases. The final data matrix consisted of 29 amino acids and 67 cases (TBM = 33, control = 34). Shifted log transformation and autoscaling was applied for multivariate analyses.

## Results

### Data quality assessment

The entire experiment was run as one batch to ensure no batch effects. EQ samples were analyzed at equal intervals throughout the run to assess the quality of the GC-MS data produced. The first EQ (EQ1) was injected four times—twice at the start (EQ1A and EQ1B) and twice at the end (EQ1C and EQ1D) of the run—to assess the stability of each analyzed amino acid over time as the entire run time of the experiment was approximately 22 h. Each sample was pre-loaded onto an autosampler at room temperature. The means of the first two repeat EQ1 samples were compared with those of the last two repeat EQ1 samples. The results of the data quality assessment are given in the Table S2. Overall, the derivatized samples remained stable while within the autosampler. The only significantly notable amino acid concentration differences were those of cystathionine, which dropped by 42%, and of cystine and leucine, which increased by 34 and 40%, respectively. However, manual inspection of the data showed that the first value of cystine (EQ1A) was extremely low (an outlier); removal of this outlier revealed that cystine changed by only 3.42% over the entire run, indicating that the associated increase was a false assessment. By examining the EQ samples over the complete run and comparing them with ERNDIM consensus values (Fowler et al., 2008), we found that asparagine and cystine fell within the expected 95% confidence intervals—indicating very good reliability (indicated by the green section in Table S2). The amino acids with poor performance (see red section in Table S2) were glutamine, leucine, ornithine, tryptophan, and valine; glutamine demonstrated the greatest variability over the experimental run. The remaining 15 amino acids (alanine, alpha-aminobutyric acid, aspartic acid, cystathionine, glutamic acid, glycine, histidine, isoleucine, lysine, methionine, phenylalanine, proline, serine, threonine, and tyrosine) fell within the lab expected ranges of the ERNDIM consensus value and therefore had good reliability (highlighted in yellow in Table S2).

### Identification of the important discriminatory amino acids

**Figure 1:**
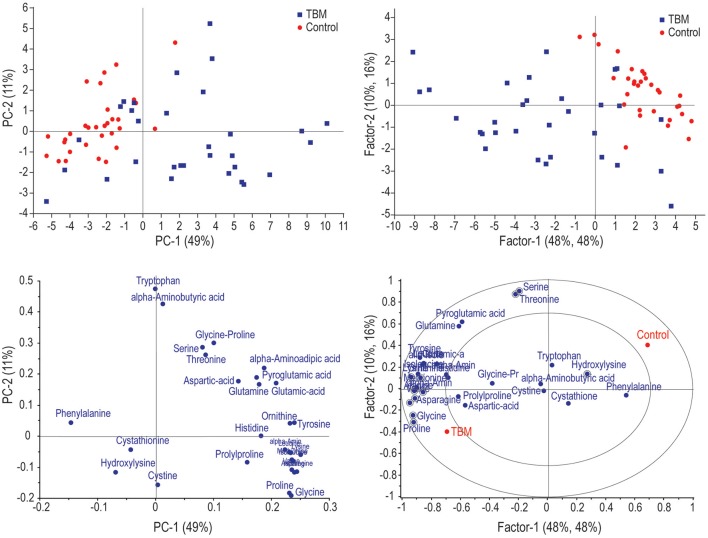
**(Left)** principal component analysis (PCA) scores plot **(top)** and corresponding loadings **(bottom)** of the various amino acids identified in the TBM and control cases. **(Right)** Partial least squares discriminant analysis (PLS-DA) scores plot **(top)** with corresponding loadings **(bottom)** indicating important discriminatory (circled) variables.

**Figure 2:**
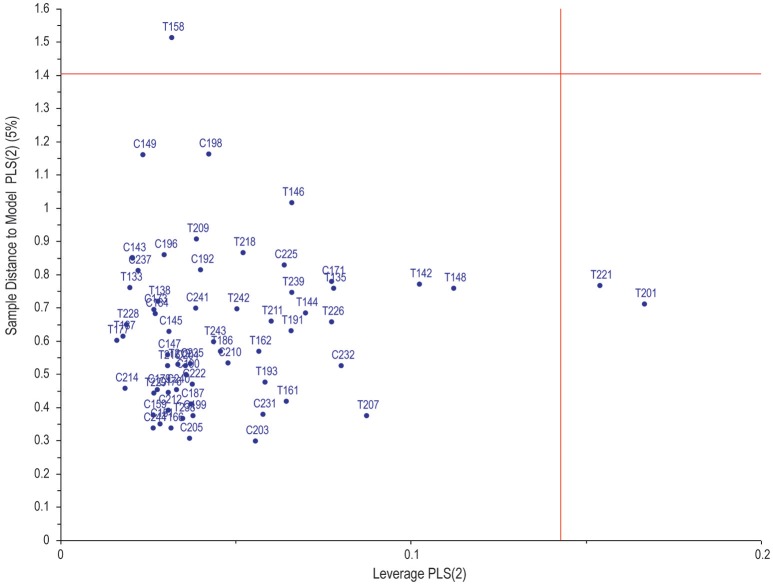
SIMCA analysis of the PLS-DA model indicating only three misclassifications for the TBM group—three false negatives predicted. Reference numbers with prefix T indicate TBM; similarly, prefix C indicates control.

Figure 1 illustrates the application of unsupervised PCA and supervised partial least squares discriminant analysis (PLS-DA). Based on these analyses, the two groups can be differentiated based purely on amino acids, but not completely separated. Within the PLS-DA correlation loadings plot there are 11 amino acids (circled on the figure)—identified as being the most important discriminatory metabolites based upon leave-one-out cross-validation (*R*^2^ = 63.9%, *Q*^2^ = 55.3%). These 11 amino acids are: alanine, alpha-aminobutyric acid, asparagine, glycine, hydroxylysine, lysine, ornithine, proline, serine, threonine, and valine. Soft independent modeling of class analogy (SIMCA) analysis was performed (Figure 2) based on the PLS-DA model, indicating three misclassifications for the TBM group. Hence, based on the prediction value of our PLS-DA model, three TBM cases were misclassified as control cases. The strength of this model is that there are no false positive classifications.

**Figure 3:**
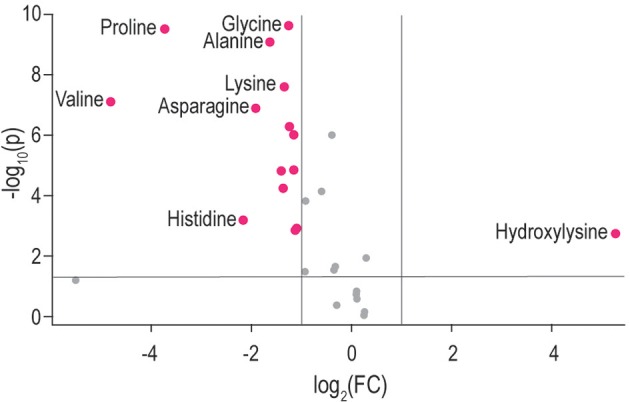
Volcano plot indicating amino acids in TBM-infected CSF based on univariate analyses—y-axis: Mann–Whitney *p*-value, x-axis: fold change. Significant amino acids with *p* < 0.05 and fold change > |1.0| are labeled; namely: alanine, asparagine, glycine, histidine, hydroxylysine, lysine, proline, and valine.

Univariate statistical analyses were also performed using Mann–Whitney *p*-values and fold changes. The results of these univariate analyses are illustrated in the volcano plot (Figure 3). Amino acids with significant values (Mann–Whitney *p* < 0.05 and fold change > 1.0) are indicated, namely, alanine, asparagine, glycine, histidine, lysine, proline, hydroxylysine, and valine.

**Figure 4:**
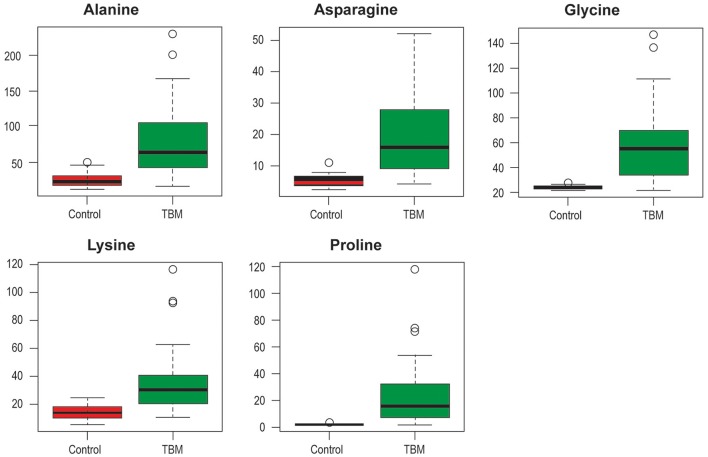
Box plots of the five most important amino acids in CSF that discriminate between TBM and controls. Y-axis indicates CSF concentration (μmol/l); ○ indicates outliers.

Based on the combination of both multivariate and univariate analyses, therefore, the most important, common, discriminatory amino acids of the control and TBM cases are: alanine (TBM: 77.3 ± 52 μmol/l, control: 25 ± 9.5 μmol/l, ref.^1^: 11–53.5 μmol/l); asparagine (TBM: 19.9 ± 14 μmol/l, control: 5.3 ± 1.7 μmol/l, ref.: 4–10 μmol/l); glycine (TBM: 58.2 ± 31.7 μmol/l, control: 24.4 ± 1.4 μmol/l, ref.: 1–10 μmol/l); lysine (TBM: 36.5 ± 25.2 μmol/l, control: 14.5 ±5.2 μmol/l, ref.: 6–37 μmol/l); and proline (TBM: 24.3 ± 25.8 μmol/l, control: 1.8 ± 0.5 μmol/l, ref.: 0–3 μmol/l) (shown as box plots in Figure 4). From the quality control assessment, these five amino acids indicate good reliability and hence are confidently identified as the important discriminatory variables for this study. It is important to note that hydroxylysine and valine were present as important amino acids but valine failed in the EQ reliability test and hydroxylysine was not part of the quality control assessment, hence neither of the last two was included in the discussion.

## Discussion

The present targeted GC-MS data correspond with our previous untargeted NMR study (Mason et al., 2015), which highlighted alanine, lysine and branched-chain amino acids as important as well as other, non-amino acid metabolites. Similar to our earlier untargeted NMR study, the five discriminatory metabolites identified here are associated with perturbed energy metabolism. As previously underscored in a review of lactate shuttles (Mason, 2017): “Understanding brain energy metabolism—neuroenergetics—is becoming increasingly important as it can be identified repeatedly as the source of neurological perturbations.” Here, alanine is closely linked to lactate and the associated shuttling systems. The data of the study presented here further highlight the importance of understanding neuroenergetics when the brain is in crisis. Also, alanine, like glycine, is an inhibitory neurotransmitter in the brain. Furthermore, as discussed in Mason et al. (2015), elevated lysine seen in CSF from TBM patients has been associated with mental retardation and in other motor neuron diseases (Shaw et al., 1995); it has also been shown to form adducts with other compounds, such as acrolein–lysine, a marker of lipid peroxidation in childhood meningitis (Tsukahara et al., 2002).

Asparagine, synthesized from central metabolic pathway intermediates, has the precursor oxaloacetic acid—an intermediate of the energy (Krebs) cycle. Asparagine is important in the metabolism of toxic ammonia in the body through the action of asparagine synthase, which attaches ammonia to aspartic acid in an amidation reaction. According to Qureshi et al. (1998), nitrites are significantly elevated in TBM cases. Hence, increased nitrogen excretion is expected in TBM patients in the form of elevated ammonia. This nitrogen excretion by-product is thus expected to be removed from the brain with asparagine as the carrier. Of special note, all five identified discriminatory amino acids are either directly or indirectly linked to ammonia. Asparagine is also involved in the glutamate-glutamine cycle, in which the enzyme asparagine synthetase produces asparagine, AMP, glutamate, and pyrophosphate from aspartate, glutamine and ATP. In the asparagine synthetase reaction, ATP is used to activate aspartate, forming beta-aspartyl-AMP. Glutamine donates an ammonium group, which reacts with beta-aspartyl-AMP to form asparagine and free AMP. Although glutamic acid and glutamine were not identified as significantly perturbed in this study, it can be postulated that a significant increase in asparagine can still be linked to a slightly perturbed glutamate-glutamine cycle in conjunction with increased ammonia—being particularly toxic in the brain as it alters neurotransmitter homeostasis. This postulate can be extended to proline, which is biosynthetically derived from glutamate and its immediate precursor, 1-pyrrole-5-carboxylate. Proline is a proteinogenic amino acid and is incorporated into proteins by prolyl-tRNA. Since elevated protein levels in CSF is a classic hallmark of TBM, it is not surprising to find proline at elevated levels. Proline is, however, used mainly for collagen formation and is important in maintaining good health in muscle, joints, and tendons. Perturbed levels of proline are normally associated with degenerative arthritis. Increased turnover of proline was, however, found in probable Alzheimer's disease cases (Fonteh et al., 2007) without arthritis, possibly reflecting increased brain degeneration.

In a study on the change in CSF amino acid profiles following an acute tonic-clonic seizure, Rainesalo et al. (2004) measured amino acid levels in CSF before and after a seizure. Rainesalo et al. reported an increase in alanine (21.5 ± 1.2 to 23.1 ± 1.4 μmol/l), asparagine (6.2 ± 0.3 to 7.7 ± 0.5 μmol/l), and glycine (5.8 ± 0.5 to 7.4 ± 0.7 μmol/l), and a slight decrease in lysine (17.7 ± 0.9 to 17.1 ± 0.9 μmol/l)—none statistically significant. Understandably, an acute tonic-clonic seizure (full body, grand mal, seizure) is a severe physiological response, yet Rainesalo et al. reported only slight changes in most amino acids (significant changes in taurine, ornithine, and phenylalanine). Table 1 compares the five discriminatory amino acids identified in our study, as being most important in TBM, with those of studies of a chronic neuroinflammatory disease, Alzheimer's, and a psychiatric disorder, schizophrenia. This comparison indicates that these particular five amino acids occur at levels 2–10 times greater in TBM, further highlighting the severity of the pathophysiological condition of this disease.

**Table 1:** Summary of concentrations of alanine, asparagine, glycine, lysine, and proline as reported for TBM in this study, compared to previously reported values for Alzheimer's and schizophrenia (concentrations given as μmol/l).

	**Alzheimer's (Fonteh et al., 2007)**	**Schizophrenia (Do et al., 1995)**	**This study (TBM)**
Alanine		4.04 ± 0.57	77.3 ± 52
Asparagine	1.77 ± 0.16	6.01 ± 0.65	19.9 ± 14
Glycine	0.28 ± 0.12	4.50 ± 0.75	58.2 ± 31.7
Lysine	44.6 ± 10.8		36.5 ± 25.2
Proline	0.68 ± 0.35		24.3 ± 25.8

## Concluding remarks

In this study, we used the sensitive method of GC-MS to profile the amino acids of CSF collected from pediatric patients classified as being “definite” or “probable” cases of TBM. Through stringent quality assessment measures and statistical analyses (univariate and multivariate) we identified five amino acids in our experimental TBM patients as being reliable and having strong discriminatory power. The concentrations of the amino acid markers of TBM here occur at levels many times greater than other neuropathological conditions, highlighting the severity of TBM. Our latest observations justify the need for more detailed studies on biochemical markers of TBM to be validated in larger-scale, and preferably multi-institutional, follow-up investigations as amino acid profiling has potential in aiding in earlier diagnosis, and hence crucial earlier treatment of TBM.

## Author contributions

All authors listed have made a substantial, direct and intellectual contribution to the work, and approved it for publication.

### Conflict of interest statement

The authors declare that the research was conducted in the absence of any commercial or financial relationships that could be construed as a potential conflict of interest.

## Footnotes

^1^Reference ranges given for children (0–10 years) obtained from the Human Metabolome Database, based upon values reported values from the University of British Columbia, B.C.'s Children's Hospital Biochemical Genetics Lab.
