# Supplementary material for: Cerebrospinal Fluid Amino Acid Profiling of Pediatric Cases with Tuberculous Meningitis
Source: Front Neurosci. 2017 Sep 26;11:534. doi: 10.3389/fnins.2017.00534 (PMC5623012; doi:10.3389/fnins.2017.00534)
Supplement: Supplementary file 1 [file DataSheet1.PDF]

Supplementary Information

Cerebrospinal Fluid Amino Acid Profiling of Pediatric Cases with Tuberculous Meningitis

Shayne Mason[1], Carolus J. Reinecke[1], Regan Solomons[2]

[1]Centre for Human Metabolomics, Faculty of Natural Sciences, Private Bag X6001, North-West University (Potchefstroom Campus), South Africa,

[2]Department of Paediatrics and Child Health, Faculty of Medicine and Health Sciences, Stellenbosch University, PO Box 19063, Tygerberg 7505, South Africa,

Table S1| Clinical information on the TBM cases and controls. [Ethnicity: b = black, m = mixed; Gender: f = female, m = male; HIV status: pos = positive, Neg = negative; CXR = chest x-ray]

Ref	Initial Diagnosis	Final Diagnosis	Ethnicity	Age	Gender	TB contact	HIV status	TBM stage admission	CSF glucose mmol/L	CXR evidence of PTB	other evidence of T
T133	TBM	Probable TBM	b	1y9m	f	y	pos	1	4,1	yes	Pos Gastric w
T135	TBM	Definite TBM	m	12y1m	f	y	neg	2b	1,2	no	no
T137	TBM	Probable TBM	m	6y11m	f	y	neg	3	4,8	no	no
T138	TBM	Definite TBM	m	3y4m	f	y	neg	1	1,2	yes	no
T142	TBM	Definite TBM	b	2y5m	f	y	neg	2b	3,5	no	Pos Gastric w
T144	TBM	Probable TBM	m	3y2m	f	y	neg	3	2,8	no	Pos Gastric w
T146	TBM	Probable TBM	m	11y3m	m	n	neg	2b	0,6	yes	not don
T148	TBM	Definite TBM	m	1y11m	f	n	neg	2a	0,5	no	negativ
T158	TBM	Probable TBM	m	2y10m	m	y	neg	1	1,8	no	Pos Gastric w
T161	TBM	Probable TBM	b	2y10m	f	y	neg	2b	0,5	no	negativ
T162	TBM	Probable TBM	m	3y10m	f	y	neg	2b	1,4	no	negativ
T166	TBM	Probable TBM	b	3y2m	m	y	neg	3	1,1	no	negativ
T177	TBM	Probable TBM	b	10m	m	y	neg	3	3,8	no	not don
T186	TBM	Probable TBM	m	2y3m	f	n	pos	2b	0,2	yes	not don
T191	TBM	Definite TBM	b	2y6m	f	n	pos	2a	0,2	yes	no
T193	TBM	Definite TBM	m	5y8m	m	n	neg	1	1,6	no	Pos Gastric w
T201	TBM	Definite TBM	b	3y1m	m	y	neg	3	3,7	yes	Pos Gastric w
T207	TBM	Definite TBM	b	4y3m	m	n	neg	2a	2,7	no	no
T209	TBM	Definite TBM	b	3y10m	f	n	neg	3	unknown	no	Trachea aspirate MT
T211	TBM	Probable TBM	m	8m	m	y	neg	3	3,9	yes	Pos sputu
T216	TBM	Definite TBM	m	3y9m	f	y	neg	2b	1,1	yes	Pos Gastric w
T218	TBM	Definite TBM	b	3y8m	m	y	pos	3	2,4	no	no
T219	TBM	Definite TBM	m	11m	f	n	neg	3	unknown	no	no
T221	TBM	Probable TBM	m	1y3m	f	y	neg	3	2,5	no	no
T226	TBM	Probable TBM	m	7y1m	m	y	neg	3	unknown	no	no
T228	TBM	Definite TBM	b	6y7m	m	n	neg	2b	5,1	no	no
T229	TBM	Probable TBM	m	11m	f	n	neg	2a	2,7	yes	Pos Gastric w
T238	TBM	Probable TBM	m	5y3m	f	n	neg	1	3,6	no	no
T239	TBM	Probable TBM	m	3y4m	f	n	neg	3	3,0	yes	Mandible pus swab M
T242	TBM	Probable TBM	m	1y3m	f	y	neg	3	0,3	no	no
T243	TBM	Probable TBM	b	3y3m	f	n	neg	2b	0,6	yes	no
[redacted]											
C143	Control	Not TBM, febrile convulsions	m	3y7m	f	y	neg	n/a	3,8	no	negativ

C145	Control	Not TBM, Kwashiorkor & PTB	m	1y9m	f	y	neg	n/a	3,5	yes	no
C147	Control	Not TBM, organophosphate poisoning	m	5y8m	m	n	neg	n/a	4,5	yes	negativ
C149	Control	Not TBM, diagnosis unknown	m	12y	f	n	neg	n/a	4,2	not done	not don
C151	Control	Not TBM, convulsions	m	1y6m	m	n	neg	n/a	3,7	yes	negativ
C159	Control	Not TBM, diagnosis unknown	w	10m	f	n	neg	n/a	4,0	not done	negativ
C170	Control	Not TBM, febrile convulsions	m	3y5m	m	n	neg	n/a	6,4	not done	not don
C171	Control	Not TBM, febrile convulsions	m	2y	f	y	neg	n/a	3,7	no	negativ
C173	Control	Not TBM, febrile convulsions	m	2y3m	m	n	neg	n/a	3,9	yes	not don
C176	Control	Not TBM, UTI	m	6m	m	y	neg	n/a	2,9	not done	Pos Gastric washing and pos
C179	Control	Not TBM, convulsions	b	4y6m	f	n	neg	n/a	3,1	no	negativ
C184	Control	Not TBM, brain abscess	b	7y4m	m	y	neg	n/a	4,6	not done	not don
C187	Control	Not TBM, hypoglycemia	b	1y2m	f	n	neg	n/a	2,3	no	not don
C190	Control	Not TBM, convulsions	b	1y2m	f	n	neg	n/a	3,9	no	not don
C192	Control	Not TBM, Kwashiorkor & PTB	m	1y11m	f	n	neg	n/a	3,3	no	Pos Gastric washing ar
C196	Control	Not TBM, diagnosis unknown	b	1y7m	f	y	pos	n/a	3,3	yes	no
C199	Control	Not TBM, hallucinations	b	3y5m	f	n	neg	n/a	unknown	no	no
C203	Control	Not TBM, bronchiolitis	m	1y4m	m	n	neg	n/a	4,0	yes	no
C204	Control	Not TBM, TB mastioditis	b	1y9m	m	y	neg	n/a	4,6	no	Right mastoid MTb
C205	Control	Not TBM, febrile convulsions	m	1y8m	m	n	neg	n/a	3,8	no	no
C210	Control	Not TBM, otitis media	b	5y4m	m	y	neg	n/a	4,9	no	no
C212	Control	Not TBM, febrile convulsions	m	2y1m	m	n	neg	n/a	3,1	no	no
C214	Control	Not TBM, tonsillitis	m	3y11m	m	n	neg	n/a	3,4	no	no
C222	Control	Not TBM, cerebral oedema	m	8y11m	m	y	neg	n/a	3,6	no	no
C225	Control	Not TBM, convulsions	b	2y7m	f	n	neg	n/a	4,1	no	no
C231	Control	Not TBM, PTB	m	4y	m	n	neg	n/a	4,2	yes	no
C232	Control	Not TBM, varicella zoster	b	5m	m	n	neg	n/a	4,0	no	no
C235	Control	Not TBM, convulsions	b	8m	f	y	pos	n/a	4,6	no	no
C237	Control	Not TBM, convulsions	b	1y6m	f	n	pos	n/a	4,4	no	no
C240	Control	Not TBM, convulsions	m	3y	f	n	neg	n/a	4,3	no	no
C241	Control	Not TBM, gastroenteritis	b	1y11m	m	n	neg	n/a	2,3	no	no
C244	Control	Not TBM, tuberculoma	b	2y7m	m	y	neg	n/a	4,3	no	no

Table S2| Concentrations (µmol/l) of ERNDIM external quality (EQ) control amino acid samples to determine data reliability. Color code (reliability): green = within 95% CI (very good); yellow = within expected lab range (good); red = outside expected range, with respect to ERNDIM consensus values.

	**Alanine**	**2-Amino-butyric-**	**Asparagine**	**Aspartic acid**	**Cysta-thionine**	**Cystine**	**Glutamic acid**	**Glutamine**	**Glycine**	**Histidine**	**Isoleucine**	**Leucine**	**Lysine**	**Methionine**	**Ornithine**	**Phenyl-alanine**	**Proline**	**Serine**	**Threonine**	**Tryptopha n**	**Tyrosine**	**Valine**
EQ1A	393.72	31.80	100.28	50.72	36.32	19.52	147.18	870.76	512.34	308.40	234.62	389.78	366.84	262.74	451.28	414.80	343.14	142.48	189.78	166.82	288.30	481.42
EQ1B	393.66	32.08	97.66	50.82	49.14	51.80	146.30	902.10	509.72	272.56	233.40	388.22	351.80	270.86	443.46	415.26	343.12	140.40	190.88	180.08	286.38	482.80
EQ2	392.60	33.90	101.10	51.46	45.00	46.36	141.36	960.76	509.20	277.10	227.88	379.74	336.24	260.60	418.58	409.86	341.82	141.88	153.04	206.78	283.20	475.88
EQ3	383.74	35.52	92.92	48.42	42.34	45.40	133.54	766.24	509.72	262.02	217.86	361.98	307.36	246.80	379.50	396.88	331.24	140.56	146.56	165.74	265.96	449.04
EQ4	351.70	33.48	85.98	43.42	13.02	41.04	119.36	743.96	475.82	223.48	196.30	326.86	279.66	215.94	331.86	362.04	304.06	130.72	133.20	150.72	235.68	405.78
EQ5	366.26	35.68	91.74	46.84	11.22	47.26	127.76	833.04	503.58	259.06	206.38	367.88	295.48	220.56	357.52	375.58	321.34	133.68	139.88	157.72	250.32	430.08
EQ6	394.14	38.96	98.98	52.16	24.28	48.40	140.32	859.18	532.42	273.48	223.70	408.38	306.36	236.96	385.22	404.20	344.60	144.42	154.42	172.16	273.66	465.82
EQ7	387.12	37.16	101.94	51.70	62.86	37.16	147.86	897.74	522.86	275.62	228.56	424.04	330.92	239.42	395.38	414.16	347.38	142.82	152.92	179.94	278.60	474.96
EQ8	369.26	36.36	95.12	47.36	41.74	48.68	129.70	724.22	506.64	261.80	211.82	398.14	289.72	217.42	365.32	384.44	324.24	132.36	137.86	160.48	251.56	435.80
EQ9	386.54	36.24	99.70	50.02	33.28	43.34	138.90	806.90	516.66	240.94	226.36	467.82	317.92	221.20	388.16	406.72	343.34	145.10	151.62	176.84	270.78	466.30
EQ10	338.86	31.42	84.86	43.26	43.46	50.06	120.44	765.00	459.56	233.82	194.96	418.52	275.50	182.50	331.10	361.02	299.30	126.18	134.08	160.64	239.92	403.64
EQ11	375.84	34.60	94.90	49.14	45.14	41.90	142.50	817.62	512.00	263.58	221.32	504.92	318.04	211.86	396.32	399.48	332.58	136.48	140.12	192.70	263.62	447.52
EQ1C	382.12	31.66	100.32	51.78	21.06	46.10	154.96	888.62	514.40	371.98	239.92	548.40	365.82	229.60	464.38	422.92	343.20	139.22	153.48	185.04	293.58	479.60
EQ1D	384.12	32.88	99.26	51.32	27.96	49.86	152.66	928.82	509.94	284.48	239.66	546.84	366.68	229.22	465.10	424.04	341.72	139.76	155.64	201.36	294.84	477.96
mean	378.55	34.41	96.05	49.17	35.49	44.06	138.77	840.35	506.78	272.02	221.62	423.68	322.02	231.83	398.08	399.39	332.93	138.29	152.39	175.50	269.74	455.47
std.dev	16.75	2.33	5.47	2.98	14.57	8.11	11.20	72.68	18.32	35.88	14.61	68.40	31.97	23.47	45.07	20.97	15.44	5.62	17.87	16.72	19.42	27.49
<u>ERNDIM consensus values</u>																						
mean	373.00	30.90	99.60	46.40	30.40	44.80	118.00	619.00	514	229	190	290	276	246	330	354	304	143	159	98.6	234	393
confidence interval (95%)	369-377	30-31	96-103	45-48	29-31	44-46	116-120	612-626	508-520	226-232	188-192	287-293	273-279	243-249	326-334	350-358	300-308	141-145	158-160	97-100	231-237	389-397
expected lab range	312-434	24-38	51-148	29-64	17-44	33-56	93-143	503-735	413-615	178-280	155-225	239-341	222-330	198-294	265-395	291-417	240-368	115-171	136-182	75-122	185-283	335-451
Reliability	yellow	yellow	green	yellow	yellow	green	yellow	red	yellow	yellow	yellow	red	yellow	yellow	red	yellow	yellow	yellow	yellow	red	yellow	red
EQ1A	393.72	31.80	100.28	50.72	36.32	19.52	147.18	870.76	512.34	308.40	234.62	389.78	366.84	262.74	451.28	414.80	343.14	142.48	189.78	166.82	288.30	481.42
EQ1B	393.66	32.08	97.66	50.82	49.14	51.80	146.30	902.10	509.72	272.56	233.40	388.22	351.80	270.86	443.46	415.26	343.12	140.40	190.88	180.08	286.38	482.80
EQ1C	382.12	31.66	100.32	51.78	21.06	46.10	154.96	888.62	514.40	371.98	239.92	548.40	365.82	229.60	464.38	422.92	343.20	139.22	153.48	185.04	293.58	479.60
EQ1D	384.12	32.88	99.26	51.32	27.96	49.86	152.66	928.82	509.94	284.48	239.66	546.84	366.68	229.22	465.10	424.04	341.72	139.76	155.64	201.36	294.84	477.96
% change over run	-2.68	1.03	0.83	1.54	-42.64	34.55	4.82	2.51	0.22	13.00	2.47	40.78	1.93	-14.01	3.88	2.04	-0.20	-1.38	-18.79	11.39	2.39	-0.69

Detailed Amino Acid SOP

EZ:faast™ Kit – Phenomenex

Free (physiological) amino acid analysis by GC-MS

Overview

The EZ:faast amino acid analysis procedure consists of a solid-phase extraction step followed by a derivatization and a liquid/liquid extraction; derivatized samples are quickly analyzed by gas chromatography–mass spectrometry. The solid-phase extraction is performed via a sorbent packed tip that binds amino acids while allowing interfering compounds to flow through. Amino acids on the sorbent tip are then extruded into the sample vial and quickly derivatized with reagent at room temperature in aqueous solution. Derivatized amino acids concomitantly migrate to the organic layer for additional separation from interfering compounds. The organic layer is then removed, evaporated, and re-suspended in re-dissolution solvent and analyzed on a GC-MS system. Total sample preparation time takes around 8 minutes and analysis is performed in around 7 minutes for a total start to finish time of approximately 15 minutes.

The EZ:faast method has been developed for the analysis of more than 60 aliphatic and aromatic amino acids, including primary and secondary amines.

Reagents

Reagent 1 (internal standard solution) – norvaline 0.2 mM + N-propanol 10%

Reagent 2 (washing solution) – N-propanol

Reagent 3A (eluting medium component 1) – sodium hydroxide

Reagent 3B (eluting medium component 2) – N-propanol

Reagent 4 (organic solution 1) – chloroform

Reagent 5 (organic solution 2) – iso-octane

NOTE: Reagents 1, 3B and 4 are stored at 4°C. Other reagents are stored at room temperature. The toxicity of all reagents is low but all steps must be taken in an exhaust hood and appropriate protection must be worn

Hexane

Equipment

Latex gloves

Sorbent tips

Sample preparation vials

Rack/holder for sample preparation vials

Plastic syringes (0.6 ml and 1.5 ml)

GC-MS autosampler vials with inserts and caps

Pipettes and pipette tips (100–1000 µl, 20–200 µl, 10–100 µl)

Drummond dialamatic microdispenser (20–100 µl)

Hamilton glass syringe (10–100 µl)

Vortex

Gas chromatograph (GC):

- 6890 GC
- Settings:
    - Temperature range: min 60°C, max 325°C
        - Increase 50°C/min until 110°C
        - Then 20°C/min until 185°C
        - Then 25°C/min until 235°C
        - Then 30°C/min until 320°C
    - Run time 11.58 min
    - Splitless mode
    - Pressure: 30.8 kPa
    - Total flow: 9.4 ml/min (carrier gas = He)
    - Injection volume: 3 µL
    - Solvent washes (hexane): 4
- GC coil/column:
    - Phenom ZB-AAA
    - 10.0 m length, 250 µm diameter, 0.25 µm film thickness
    - Max temperature: 320°C
    - Constant flow: avg 92 cm/sec

Mass spectrometer (MS):

- MS source: 240°C (max 250°C)
- MS quad: 180°C (max 200°C)
- Scan range: 40–450 m/z
    - Low mass: 40.0
    - High mass: 450.0
    - Threshold: 100
- EM voltage: 2023.5
- Solvent delay: 2.50 min

Sample Preparation:

Sample types: serum, urine, cerebrospinal fluid (CSF)

1. For each sample, line up one glass sample preparation vial in the vial rack.
2. Pipette sample (100 µl urine or 50 µl plasma/serum) and 100 µl Reagent 1 (internal standard solution) into each sample preparation vial.
3. Attach a sorbent tip to a 1.5 ml syringe and loosen the syringe piston; immerse the tip and let the solution in the sample preparation vial pass through the sorbent tip by slowly pulling back the syringe piston, in small steps.
4. Pipette 200 µl Reagent 2 (washing solution) into the same sample preparation vial. Pass the solution slowly through the sorbent tip and into the syringe barrel. Drain the liquid from the sorbent bed by pulling air through the sorbent tip. Detach the sorbent tip, and leave it in the sample preparation vial, then discard the liquid accumulated in the syringe.
5. Prepare the eluting medium as follows:

Number of samples	Reagent 3A eluting medium Component 1	Reagent 3B eluting medium Component 2
2	300 µl	200 µl
4	600 µl	400 µl
7	900 µl	600 µl
12	1.5 ml	1.0 ml
14	1.8 ml	1.2 ml
19	2.4 ml	1.6 ml

24	3.0 ml	2.0 ml
29	3.6 ml	2.4 ml
34	4.2 ml	2.8 ml

6. Pipette 200 µl freshly prepared eluting medium into same sample preparation vial.
7. Pull back the piston of a 0.6 ml syringe halfway up the barrel and attach the sorbent tip used in steps 3–8.
8. Wet the sorbent with the eluting medium; watch as the liquid rises through the sorbent particles and stops when the liquid reaches the filter plug in the sorbent tip.
9. Eject the liquid and sorbent particles out of the tip and into the sample preparation vial. Repeat steps 7 and 8 until the sorbent particles in the tip are expelled into the sample preparation vial.
10. Pipette 50 µl Reagent 4 (organic solution 1), using Drummond microdispenser, into the sample preparation vial.

NOTE: clean the Drummond microdispenser after each analysis by collecting approximately 50 µl 70% ethanol solution (70 ml absolute ethanol diluted to 100 ml with distilled water) followed by rinsing with distilled water.

11. Emulsify the liquid in the vial by vortexing for 5–8 seconds, allow to stand for 1 min (perform step 11 three times).
12. Pipette 100 µl Reagent 5 (organic solution 2), using Drummond microdispenser, into the sample preparation vial and repeat vortexing procedure in step 11.

NOTE: clean the Drummond microdispenser after each analysis by collecting approximately 100 µL 70% ethanol solution followed by rinsing with distilled water.

13. After allowing the reaction to proceed for 1 min, transfer part of the (upper) organic layer (about 50–100 µl) using a Hamilton glass syringe into an autosampler vial for GC-MS analysis.

NOTE: clean the Hamilton glass syringe after each transfer by collecting approximately 100 µl hexane and discarding (repeat 5 times).

NOTE: all organic waste to be discarded into designated organic waste container.
